# A FEM-Based Optimization Method for Driving Frequency of Contactless Magnetoelastic Torque Sensors in Steel Shafts

**DOI:** 10.3390/ma14174996

**Published:** 2021-09-01

**Authors:** Anna Ostaszewska-Liżewska, Michał Nowicki, Roman Szewczyk, Mika Malinen

**Affiliations:** 1 Faculty of Mechatronics, Institute of Metrology and Biomedical Engineering, Warsaw University of Technology, sw. A. Boboli 8, 02-525 Warsaw, Poland; michal.nowicki@pw.edu.pl; 2 Łukasiewicz Research Network—Industrial Research Institute for Automation and Measurements PIAP, 02-486 Warsaw, Poland; roman.szewczyk@pw.edu.pl; 3 CSC–IT Center for Science, P.O. Box 405, FI-02101 Espoo, Finland; mika.malinen@csc.fi

**Keywords:** contactless torque sensors, magnetic permeability tensor, torque sensor, FEM optimization

## Abstract

This paper presents a novel finite element method (FEM) of optimization for driving frequency in magneto-mechanical systems using contactless magnetoelastic torque sensors. The optimization technique is based on the generalization of the axial and shear stress dependence of the magnetic permeability tensor. This generalization creates a new possibility for the determination of the torque dependence of a permeability tensor based on measurements of the axial stress on the magnetization curve. Such a possibility of quantitative description of torque dependence of a magnetic permeability tensor has never before been presented. Results from the FEM-based modeling method were validated against a real magnetoelastic torque sensor. The sensitivity characteristics of the model and the real sensor show a maximum using a driving current of similar frequency. Consequently, the proposed method demonstrates the novel possibility of optimizing magnetoelastic sensors for automotive and industrial applications.

## 1. Introduction

Measuring the torque in mechanical shafts made of steel is a common challenge found in various industrial applications. Some examples of these applications include marine propulsion systems [1,2], turbine generators [3,4,5], robotics [6,7,8,9,10], and automotive [11,12,13,14] and electromobility applications [15,16,17]. Due to the rotation of a mechanical shaft under normal working conditions, powering a typical strain-gauge based torque sensor while collecting data is very challenging [3]. For this reason, the most promising solution from an application point of view after comparing the different types of shaft torque sensors [1,18,19,20,21,22,23,24,25] is contactless sensors.

Soft magnetic materials the most commonly used as cores of contactless torque sensors utilizing magnetoelastic effects [26,27,28,29] are constructional steels [30,31,32]. However, research on the magnetoelastic effect caused by torque and possibilities of sensor optimization are significantly limited. Although the magnetoelastic effect caused by axial stresses is well described in the literature [33,34], information on the influence of shearing stresses generated by torque on magnetic characteristics of constructional steels is not readily available.

This paper addresses the gap in the state of the art by presenting the modeling results of the torque dependence of magnetic characteristics on drive shafts made of constructional steel. Following on from the previous work on the generalization of the axial and shear stress dependence of magnetic permeability tensors carried out in [35], this paper presents a novel approach to quantitative description of the torque dependences of magnetization process. Due to tensor description of magnetic permeability, the proposed approach enables considering the shear stress induced anisotropy of constructional steel magnetic permeability. Moreover, in the proposed model, the shear stress-induced anisotropy can be determined on the base of measurements of axial stress dependence of the magnetization curve [36]. This feature was not presented before in the literature. It should be highlighted that the possibility of determination of shear stress dependence of magnetic permeability tensor on the base of axial stress permeability dependence measurements is very useful from the practical point of view.

As a result, the proposed model may be efficiently applied for FEM-based modeling solutions. On the base of experimentally determined axial stress permeability dependences, the proposed model provides insight for understanding of magnetization processes under the influence of torque, as well as enables further contactless magnetoelastic torque sensors development. This development creates the possibility of optimizing magneto-mechanical systems using contactless magnetoelastic sensors in the area of modern industrial applications.

## 2. Finite Element Model for Development of Contactless Torque Sensors Utilizing Ferromagnetic Construction Steels

The description of the stress tensor $\hat{\sigma }$ dependence of relative magnetic permeability $\hat{\mu_{r}}$ tensor is the foundation of partial differential equation-based models of the magnetoelastic effect. Knowledge on the shear stress dependence of permeability is limited to specific systems and materials [37]. Moreover, axial and shear stress may occur in magneto-mechanical systems simultaneously, which makes its description more complex.

It should be highlighted that, in the presence of stress induced anisotropy of magnetic permeability in materials, flux density *B* is not parallel to magnetizing field *H*. This phenomenon was neglected before [38]. In addition, due to sophisticated mechanical stress distribution as well as due to the simultaneous presence of axial and shear stress, only finite elements modelling (FEM) methods enable the comprehensive analysis of the stress dependence of the magnetization process in real systems.

Experimental results widely presented in the literature are mostly focused on the axial stress dependence of isotropic magnetic materials such as construction steels [38,39]. The axial stress dependence of relative permeability was previously estimated as a linear dependence given as follows [40]:

(1) $$\mu_{r}\left(\sigma \right)=769+4.22\;\sigma \;$$

where *σ* and *μ_r_* represent the axial stress (expressed in MPa) and relative permeability, respectively. However, it should be highlighted that a good agreement of Equation (1) with determination coefficient *R*^2^ exceeding the value 0.94 was observed only for limited values of axial mechanical stresses *σ*.

For the generalization of stress tensor dependence of relative magnetic permeability $\hat{\mu_{r}}$ tensor, the principal stress concept was applied [35]. Considering the Mohr’s circle concept [41,42], each stress tensor can be reduced to the vector of axial stresses (principal stresses tensor $\hat{\sigma_{P}}$) that is rotated to the new coordinate system by the rotation matrix $\hat{R}$ [36,43]:

(2) $$\hat{\sigma }=\left[\begin{array}{ccc} \sigma_{xx} & \tau_{xy} & \tau_{xz}\\ \tau_{xy} & \sigma_{yy} & \tau_{yz}\\ \tau_{xz} & \tau_{yz} & \sigma_{zz} \end{array}\right]=R\times \left[\begin{array}{ccc} \sigma_{Px} & 0 & 0\\ 0 & \sigma_{Py} & 0\\ 0 & 0 & \sigma_{Pz} \end{array}\right]\times R^{-1}$$

Theoretical and experimental analyses presented in previous work indicate that effective axial stresses *σ_eff_* caused by the perpendicular stress $\sigma_{⊥}$ can be calculated according to the following dependence [42]:

(3) $$\sigma_{eff}=-\nu \times \sigma_{⊥}$$

where ν is Poisson’s ratio. The Poisson’s ratio of constructional steels is typically equal to 0.3.

Considering Equation (3), the efficient axial stresses influencing the relative magnetic permeability $\hat{\mu }$ tensor can be estimated as:

(4) $$\sigma_{eff\;Px}=\sigma_{Px}-\nu \sigma_{Py}-\nu \sigma_{Pz}$$

(5) $$\sigma_{eff\;Py}=\sigma_{Py}-\nu \sigma_{Px}-\nu \sigma_{Pz}$$

(6) $$\sigma_{eff\;Pz}=\sigma_{Py}-\nu \sigma_{Px}-\nu \sigma_{Py}$$

Consequently, the relative magnetic permeability $\hat{\mu_{r}}$ tensor can be calculated using the following equation:

(7) $$\hat{\mu_{r}}=R\times \left[\begin{array}{ccc} \mu_{r}\left(\sigma_{eff\;Px}\right) & 0 & 0\\ 0 & \mu_{r}\left(\sigma_{eff\;Py}\right) & 0\\ 0 & 0 & \mu_{r}\left(\sigma_{eff\;Pz}\right) \end{array}\right]\times R^{-1}$$

For the plain torque, only shear stress occurs in the stress tensor. In this case, Equation (2) can therefore be written as:

(8) $$\left[\begin{array}{ccc} 0 & 0 & \tau_{xz}\\ 0 & 0 & \tau_{yz}\\ \tau_{xz} & \tau_{yz} & 0 \end{array}\right]=R\times \left[\begin{array}{ccc} \sigma_{\mathrm{Px}} & 0 & 0\\ 0 & \sigma_{\mathrm{Py}} & 0\\ 0 & 0 & 0 \end{array}\right]\times R^{-1}$$

Equation (7) is thus reduced to:

(9) $$\hat{\mu_{r}}=R\times \left[\begin{array}{ccc} \mu_{r}\left(\sigma_{Px}-\nu \sigma_{Py}\right) & 0 & 0\\ 0 & \mu_{r}\left(\sigma_{Py}-\nu \sigma_{Px}\right) & 0\\ 0 & 0 & \mu_{r}\left(-\nu \sigma_{Px}-\nu \sigma_{Py}\right) \end{array}\right]\times R^{-1}$$

Plain torque is rarely observed in real systems. For this reason, it is highly recommended to implement the Finite Element Modeling (FEM) systems where the stress tensor dependence of the relative magnetic permeability tensor is based on Equation (7).

## 3. Implementation of Proposed Model in Open-Source FEM-Oriented Software

The proposed model is implemented using an open-source software with GNU licenses (Version 3, Free Software Foundation, Boston, MA, USA) which can be adapted easily by small companies without access to expensive licenses. Other commercial software such as ANSYS or COMSOL can be employed to implement the proposed optimization method of stress sensors. In this investigation, the following software were used under the LINUX MINT [44] operating system—Octave 5.2.0 [45] (open-source MATLAB alternative), NETGEN [46], Elmer FEM [47] and ParaView [48]—to establish a modeling environment.

**Figure 1:**
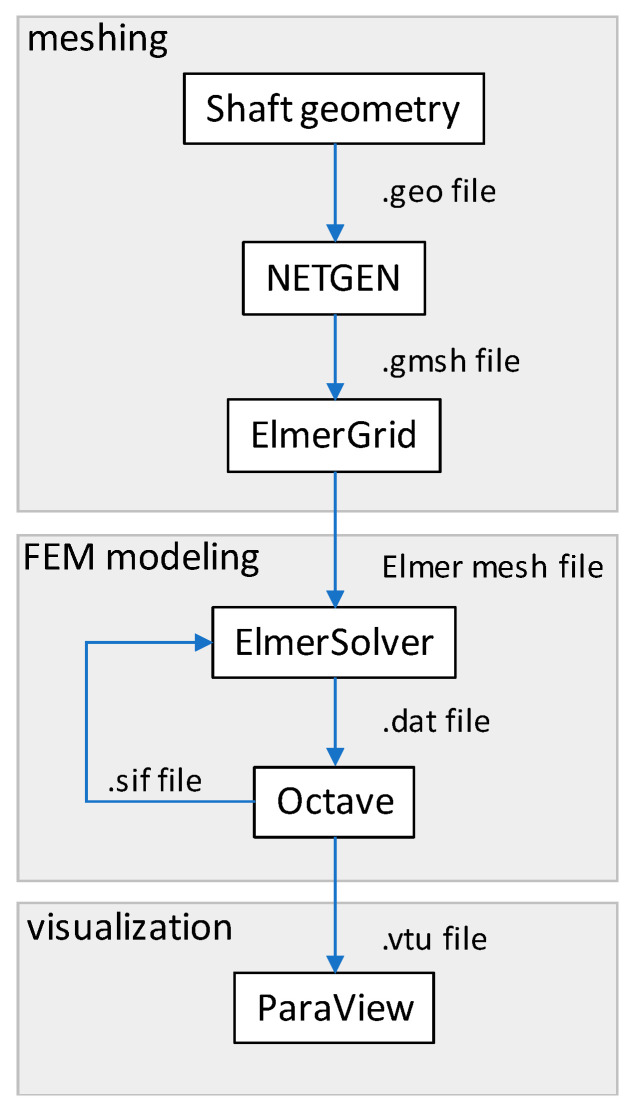
A schematic block diagram of information flow during the modeling process.

A schematic block diagram of the software interoperation is presented in Figure 1. ElmerGrid and ElmerSolver are modules of Elmer FEM software (Version 9.0, CSC—IT Center for Science, Espoo, Finland) which can be used independently. The geometry model is described in the NETGEN batch .geo file. The mesh is then computed using the Delaunay method and processed in ELMERGrid from a .gmsh file into Elmer mesh files. It should be stated that information exchange among modules is carried out using text files. These tools are beneficial for simplifying the troubleshooting and validation stages of this process.

**Figure 2:**
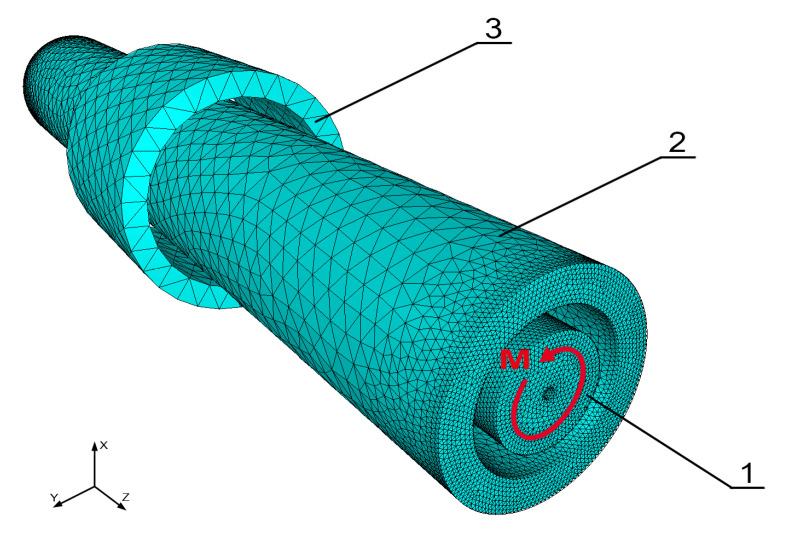
The tetrahedral mesh of the modelled system; 1—shaft, 2—magnetizing coil, 3—sensing coil.

The tetrahedral mesh of a magneto-mechanical system is presented in Figure 2. This mesh consists of a modelled shaft (1), a driving coil (2), and a sensing coil (3). The dimensions of the bounding box of the shaft with driving and sensing coils are *x* = *y* = 7.5 mm and *z* = 64 mm. The whole model was modelled in a sphere of radius *r* = 1 m. The mesh was built from 1,454,671 volume elements, 93,468 surface elements, and 140,202 edge elements in total. The sphere was assigned the properties of air at room temperature, and the copper and shaft coils were assigned the properties of structural steel. The relative magnetic permeability tensor $\hat{\mu_{r}}$ was calculated by using the procedure written in the FORTRAN programming language. The current density in the magnetizing coil and the torque load applied to the shaft were implemented within a .sif file in the MATC language as a source and boundary conditions for the model.

The output signal on the real sensor is the voltage measured across a precise 1 kΩ resistor that is connected to the sensing coil. In the FEM modeling, the voltage was calculated from the base value of the current in the sensing coil while the resistance was modelled by measuring the resistance of a whole coil. This approach ensures the actual voltage drop across the resistor can be measured accurately.

The calculations were automated using scripts in Octave which generated batch .sif files and were run using the ElmerSolver. A total of 18 simulations were carried out for nine values of supply current with AC frequencies: 50 Hz, 250 Hz, 500 Hz, 1 kHz, 1.5 kHz, 2 kHz, 3 kHz, 4 kHz, and 5 kHz. The current amplitude used was 0.5 A for two values of torque load: 1.3 Nm and 0 Nm (no load), respectively. The results were then visualized using the ParaView software.

**Figure 3:**
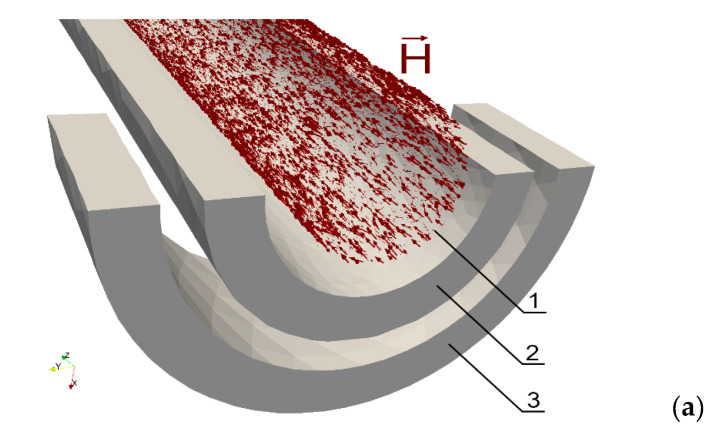

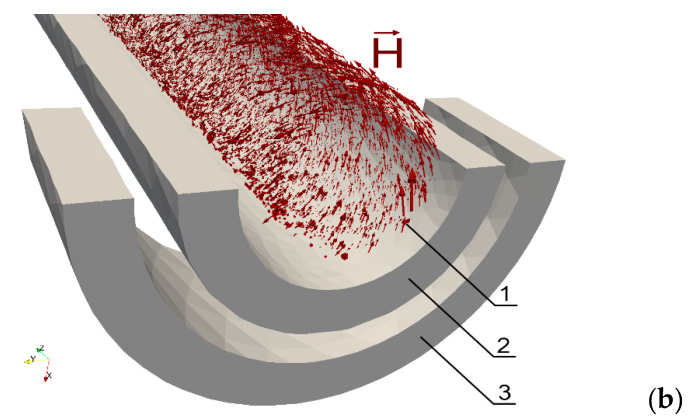
Magnetic field strength inside the shaft (first cross-section along the shaft axis and the second perpendicularly to the shaft axis, through the center of the length of the shaft and the coils) for different values of torque *M*: (**a**) *M* = 0 Nm, (**b**) torque *M* = 1.3 Nm; 1—shaft (transparent), 2—magnetizing coil, 3—sensing coil.

**Figure 4:**
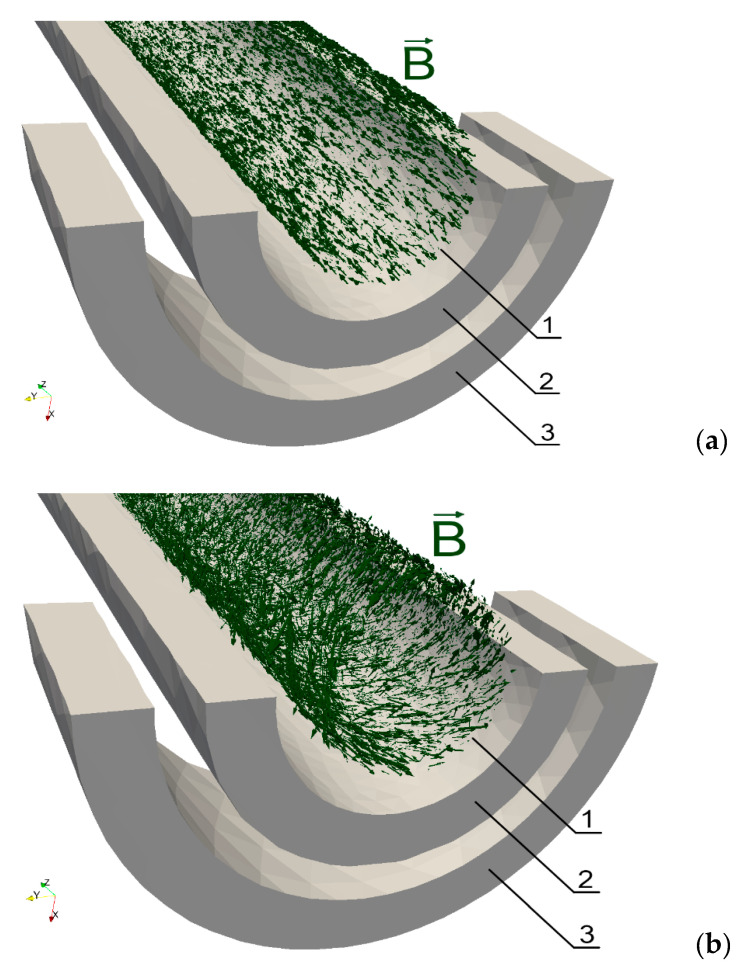
Magnetic flux density inside the shaft for different values of torque *M*: (**a**) *M* = 0 Nm, (**b**) torque *M* = 1.3 Nm; 1—shaft (transparent), 2—magnetizing coil, 3—sensing coil.

Figure 3 shows the vectors of magnetic field strength H before (Figure 3a) and after (Figure 3b) applying torque when the driving coil current frequency is equal to 5 kHz, the amplitude is 0.5 A, and the number of turns of the driving coil is 150. It is clearly seen that the direction of the vectors which were previously parallel to the axis of the shaft have now become helically skewed. The same phenomenon can be observed for magnetic flux density vectors B as shown in Figure 4 where the driving current frequency, amplitude, and number of turns of the driving coil are set to 5 kHz, 0.5 A, and 200 respectively.

The skewness of the magnetic field strength and flux density is connected with the direction of the easy axes of the stress induced anisotropy tensor $\hat{\mu_{r}}$. These easy axes are observed by the rotation matrix R which was available during the Elmer Solver modeling. It should be highlighted that the observed results of the magnetic field strength and flux density modeling agree with previously presented experimental results in references [29,49].

**Figure 5:**
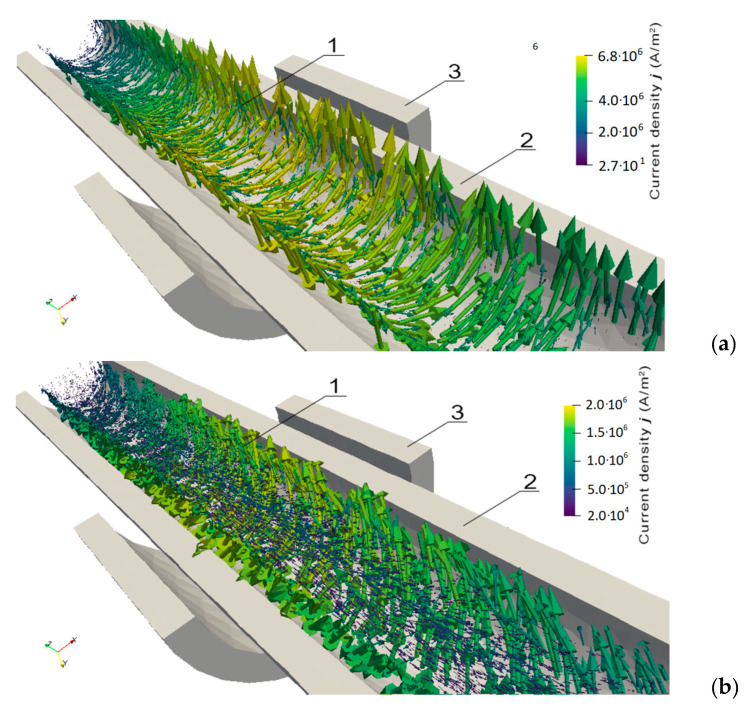
A cross-section view of the eddy current vectors along the shaft’s axis for different values of torque *M*: (**a**) *M* = 0 Nm, (**b**) torque *M* = 1.3 Nm; 1—shaft (transparent), 2—magnetizing coil, 3—sensing coil.

Figure 5a,b show a cross-section view of the eddy current densities j before and after applying torque of 1.3 Nm. Similar to the modeling conditions presented in Figure 3 and Figure 4, the driving current frequency, amplitude, and number of turns of the driving coil are 5 kHz, 0.5 A, and 150, respectively.

Before applying any torque, the current density vectors are tangent to the circle defining the circumference of the shaft and are contained in a plane perpendicular to the shaft axis. After applying a torque of 1.3 Nm, the eddy currents density vectors appearing on the shaft’s surface have a greater value and are tangent to the thread line. Looking at the whole volume excluding the surface, there are smaller vectors with a direction that is parallel to the shaft axis running in the opposite way. This shows how the current circulates on the surface and returns to the center of the shaft.

It should be highlighted that eddy current flow in the parallel direction of the shaft subjected to torque has never been observed before. In spite of the fact that the amplitude of such parallel eddy currents is relatively small, this phenomenon may be practically utilized in the area of non-destructive testing of conductive shafts subjected to torque and shear stresses. Material discontinuities inside of a shaft may influence parallel eddy current distribution, which may be observed utilizing an eddy current tomography [50]. In such a case, the model presented in Section 2 creates the possibility of quantitative description of eddy current distribution and efficient estimation of a size and character of possible discontinuities in the core of a driving shaft subjected to torque.

The cause of all the results presented in Figure 3, Figure 4 and Figure 5 is the influence of the complex stress distribution introduced by the torsion on the magnetic permeability $\hat{\mu_{r}}$ tensor, which changes the anisotropy direction of the material [36]. For further validation, the source code for modeling the magnetoelastic torque sensors is available at Supplementary Materials: https://github.com/ostaszewska/ElmerFEM_Shaft_torque under the open-source MIT license.

## 4. Experimental Validation of Proposed Model

**Figure 6:**
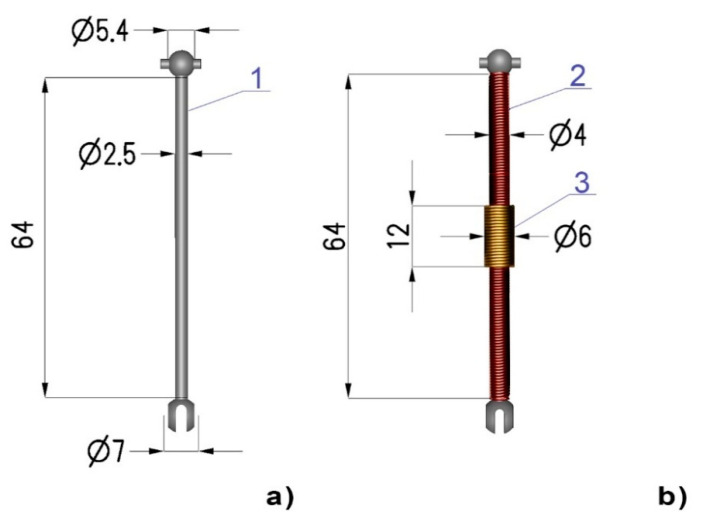
Geometry of the tested shaft: (**a**) shaft dimensions, (**b**) coil arrangement diagram; 1: shaft, 2: driving coil, 3: sensing coil. All dimensions are given in mm.

The experimental validation of the proposed model was performed on a 7250R drive shaft (Traxxas, McKinney, TX, USA) which is made of constructional steel. This type of shaft is generally used for electric vehicle models on a 1:16 scale. The geometry and dimensions of the shaft are shown in Figure 6a. For the purpose of this experiment, two coils were wound on the shaft: the magnetizing coil (where the length of the entire shaft is 150 turns) and a sensing coil of 50 turns. The coil arrangement diagram is presented in Figure 6b where numbers ‘1’, ‘2’, and ‘3’ denote the shaft, magnetizing coil, and sensing coil, respectively, and the given dimensions are in millimeters. One end of the shaft was anchored, and torque was applied to the other end by hanging weights on a lever arm.

**Figure 7:**
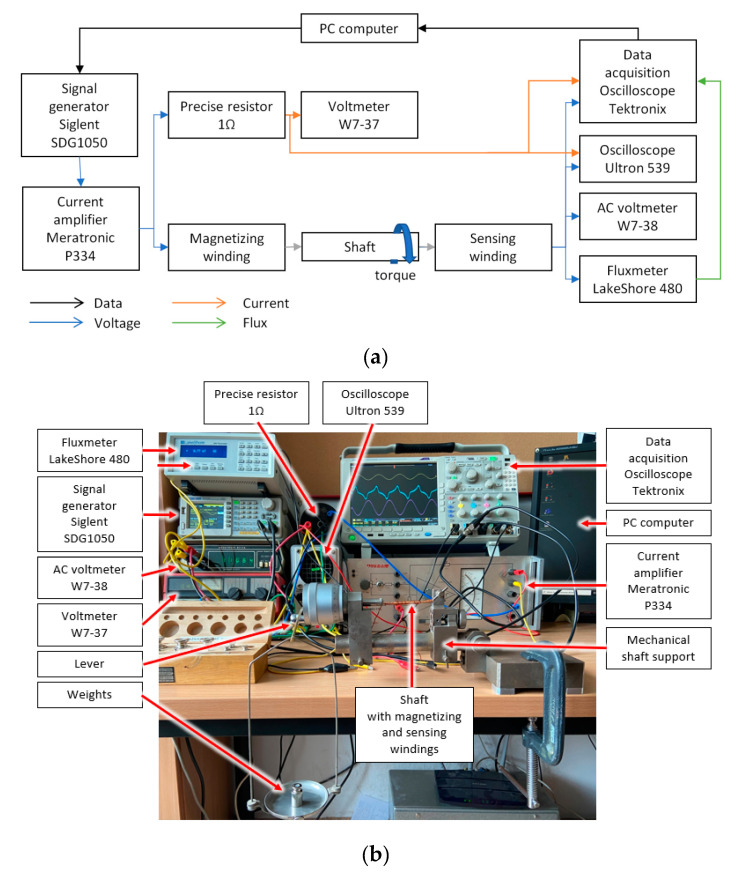
The measurement setup for testing the magnetoelastic torque sensing system: (**a**) schematic diagram, (**b**) photograph of the system.

All measurements were done with the measurement system setup which is presented in the schematic block diagram shown in Figure 7. The experimental setup consists of the following:

- SDG1050 signal generator (Siglent, Helmond, The Netherlands);
- P334 current amplifier (Meratronic, Warsaw, Poland);
- Precise resistor 1 kΩ (INCO, Warsaw, Poland);
- Voltmeter W7-37 (Petersburg, Russia);
- Fluxmeter LakeShore 480 (Lake Shore Cryotronics, Inc., Westerville, OH, USA);
- AC voltmeter W7-38 (Leningrad, USSR);
- Oscilloscope Ultron 539 (Munchen, Germany);
- Oscilloscope Tektronix MDO4024C (Beaverton, OR, USA);
- PC computer (X-KOM, Warsaw, Poland).

A dedicated software was installed on the PC computer to control the signal generator and collect data simultaneously in the LabView environment (National Instruments, Austin, TX, USA).

The experiment was carried out using 33 frequency values ranging from 50 Hz up to 5 kHz that were increased gradually in an adaptive pattern. The amplitude of driving current was constant and set to 0.5 A_Peak_. For each frequency change, the voltage induced on the sensing coil was measured for the shaft without torque (*U*_detM0_(*f*)) and when the torque value was equal to 1.3 Nm (*U*_detM1.3_(*f*)). Two values of torque M were selected to linearize the torque dependence of the output signal and to simplify the model for phenomenon explanation. The sensitivity value *S*(*f*) for each frequency was calculated according to the following equation:

(10) $$S\left(f\right)=\frac{∆U_{\det}\left(f\right)}{∆U_{\det\_range}}$$

where:

(11) $$∆U_{\det}\left(f\right)=U_{\mathrm{detM}0}\left(f\right)-U_{\mathrm{detM}1.3}\left(f\right),$$

and

(12) $$∆U_{\det\_range}=∆U_{\det\_max}-∆U_{\det\_min}$$

In Equation (12), $∆U_{\det\_max}$ is the maximum computed value of $∆U_{det}\left(f\right)$ and $∆U_{\det\_min}$ is the minimum value.

**Figure 8:**
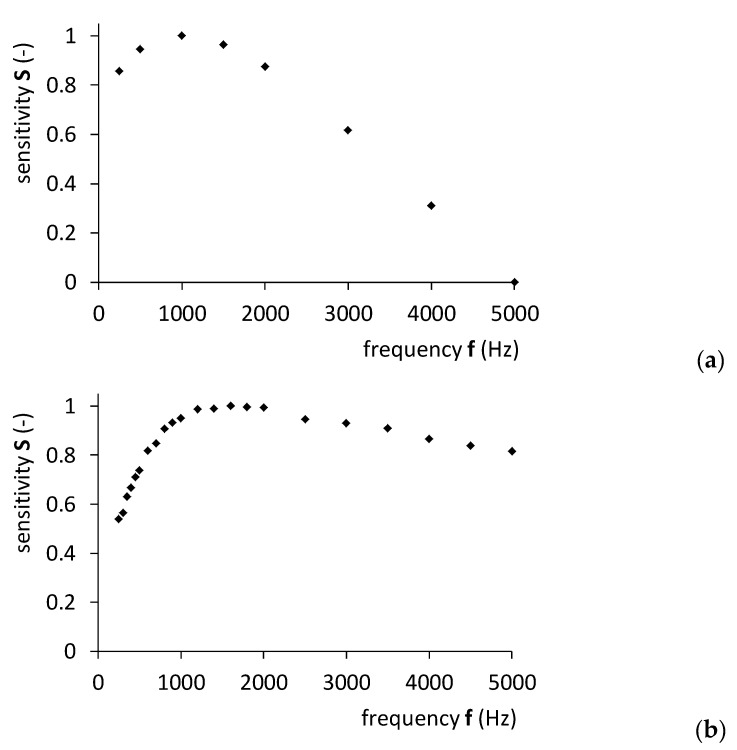
Frequency dependence of sensitivity of magnetoelastic torque sensing system: (**a**) sensitivity characteristics of the FEM model, (**b**) experimentally obtained sensitivity of the real system with 7250R shaft.

Figure 8 presents the experimental results obtained for the sensitivity value *S*(*f*) in comparison to the FEM modeling, while Table 1 provides detailed experimental results.

The output voltage and flux density characteristics were similar, which is due to low magnetizing fields used, and thus the steel shaft was working in the initial part of the magnetization curve. For practical engineering applications, the output voltage sensitivity characteristic is further discussed.

It should be highlighted that stress dependence of magnetic properties of materials (such as magnetic permeability tensor) is nonlinear for a wide range of axial or shear stresses. This nonlinearity is the main reason for differences between the results of experiments and modeling presented in Figure 8. Moreover, the decrease in magnetoelastic sensitivity for higher frequencies is connected with the strong influence of eddy current skin effect in conductive bulk elements made of constructional steel. This phenomenon is especially visible in the case of real shafts. For driving frequencies exceeding 5 kHz, the sensitivity of the sensor radically degrades due to the roughness of a surface of material and non-uniform distribution of surface stresses.

On the other hand, in both scenarios, the maximum sensitivity is observed to be approximately 1.2 kHz. As a result, in determining the optimal sensor driving frequency, important similarities can be observed between the obtained experimental results and the proposed FEM-based modeling results.

## 5. Conclusions

The presented results in this paper have confirmed the possibility of modeling the torque dependence of magnetization processes in steel shafts. Considering previously presented work on the generalization of stress dependence of a magnetic permeability tensor, the proposed generalization represents the dynamic magnetization process that is connected to the eddy currents in a magnetized shaft. From modeling, it is achievable to observe the helical anisotropy in the shaft that is subjected to torque and the axial eddy currents in this shaft. It should be highlighted that research on the axial component in eddy current distribution in magnetoelastic shafts that are subjected to torque has not been presented previously in the literature.

The FEM-based modeling results have confirmed the prospect of determining the optimal driving frequency for magnetoelastic torque sensors with a core made of construction steel. The modeling and experimental test results both showed an optimal frequency of approximately 1.2 kHz. It is noted, however, that the optimal driving frequency of magnetoelastic sensors may be strongly dependent on its geometry and the type of steel used for the shaft. Therefore, modeling should be carried out specifically for any given material using the specific geometry of the magnetoelastic sensing shaft.

In future works, better modelling accuracy could be achieved with the use of an experimentally obtained model of the Anhysteretic Magnetization curve for given material, due to the recent achievements in this area [51].

## Figures and Tables

**Table 1:** Experimentally obtained frequency dependence of flux density and output voltage with 7250R shaft.

Torque [Nm]	0		1.3	
Frequency [Hz]	Voltage [mV]	Flux Density [mT]	Voltage [mV]	Flux Density [mT]
50	11.2	101.57	9.54	86.6
60	13.29	100.59	11.38	85.91
70	15.36	99.55	13.17	85.24
80	17.38	98.63	14.9	84.6
90	19.39	97.79	16.68	83.92
100	21.37	96.94	18.4	83.28
120	25.24	95.42	21.78	82.07
140	29	93.93	25	80.88
160	32.64	92.47	28.2	79.71
180	36.16	91.06	31.3	78.59
200	39.57	89.7	34.2	77.48
250	47.6	86.5	41.4	74.98
300	55.25	83.63	48.1	72.63
350	62.37	81.01	54.44	70.52
400	69.12	78.64	60.42	68.55
450	75.49	76.43	66.1	66.73
500	81.57	74.37	71.5	65.04
600	92.8	70.69	81.6	61.98
700	103.26	67.45	90.9	59.26
800	112.8	64.57	99.54	56.82
900	121.7	61.99	107.5	54.62
1000	130.1	59.66	115	52.64
1200	145.5	55.64	128.8	49.16
1400	159.5	52.28	141.2	46.22
1600	172.3	49.44	152.6	43.72
1800	184.3	47.02	163.2	41.57
2000	195.6	44.92	173.2	39.7
2500	221.9	40.74	196.1	35.96
3000	245.7	37.6	217	33.14
3500	268	35.15	236.5	30.94
4000	289	33.18	254.6	29.176
4500	309.3	31.55	272.2	27.72
5000	328.7	30.18	289	26.49

## Data Availability

Not applicable.

## Supplementary Materials

The following are available online at https://github.com/ostaszewska/ElmerFEM_Shaft_torque.
